# Phaco Prechop versus Divide and Conquer Phacoemulsification: A Prospective Comparative Interventional Study

**DOI:** 10.4103/0974-9233.51987

**Published:** 2008

**Authors:** Effat A. Elnaby, Omar M. El Zawahry, Ahmed M. Abdelrahman, Hany E. Ibrahim

**Affiliations:** From the Department of Ophthalmology, Cairo University, Giza, Egypt

**Keywords:** karate prechop, phacoemulsification, nucleus, counter prechop

## Abstract

**Purpose:**

To compare two phaco techniques, namely Phaco Prechop and Divide and conquer, basically during their early learning curves.

**Patients and Methods:**

The study included 50 patients divided into 2 groups, each including 25 patients; group (A) where phaco Prechop was performed, and group (B) in which divide and conquer was performed. The mean effective ultrasound time, mean endothelial cell count, mean endothelial cell loss, corneal thickness, intraoperative complications, and the best corrected visual acuity were reported in the two groups both preoperative and postoperative.

**Results:**

The mean effective ultrasound time in group A was 19.36 ± 8.51 seconds, and in group B, it was 24.44 ± 7.86 seconds with a statistically significant difference between the two groups (P = 0.033). The mean endothelial cell count 3 months postoperative in group A was 2139.88 cells/mm^2^. In group B, the mean endothelial cell count 3 months postoperative was 2087.08 cells/mm^2^. The difference between the two groups was statistically insignificant (P = 0.558), however The difference in endothelial cell loss 3 months postoperatively between the two groups was statistically significant. (P = 0.001). Four cases in groups A (16%) had posterior capsular rents compared to three cases (12 %) in group B. Postoperative best corrected visual acuity in group B was 6/12 or better in 88% of cases as compared to 92% in group A with no statistical difference.

**Conclusion:**

Early cataract surgical cases performed with the Phaco Prechop and divide and conquer techniques showed comparable results and complications. However the former technique utilized less phaco time and energy without significant effect on the final surgical outcome.

Divide and Conquer is the parent nucleofracture technique. Since its original description in 1985, it has been constantly improved and numerous variations have developed. The technique was developed by Gimbel to facilitate subdivision of the nucleus into small pieces so that they could be removed more efficiently. Four basic steps are incorporated in divide and conquer technique; deep sculpting until a fracture is possible, nucleofractis of the nuclear rim and posterior plate of the nucleus, fracturing again and breaking away a wedge-shaped section of nuclear material for emulsification, and rotation or repositioning of the nucleus for further fracturing and emulsification.^1^

Phaco prechop is a nuclear fracture technique that is performed under viscoelastic material prior to phacoemulsification. Using this procedure, the surgeon can divide the nucleus without grooving or sculpting. This technique has been first introduced in 1993; however, due to fear of breaking the posterior capsule or ciliary zonules, many surgeons hesitated to attempt this procedure, or tried but abandoned the technique before acquiring the necessary level of skill.^2^

Considering the three-dimensional structure of lens fibers, longitudinal division of the nucleus is most reasonable. Akahoshi in 1993 divided the nucleus manually into four pieces before phaco. Once the nucleus has been divided, phacoemulsification can be performed easily and rapidly, even as a single-handed technique. With a high vacuum and high flow setting; each divided nuclear fragment is aspirated and phacoemulsified one by one.^3^

The aim of this prospective comparative study is to compare two phaco techniques, namely Phacoprech and Divide and conquer basically during their early learning curves.

## Patient and Methods

The study included 50 patients divided into 2 groups, each including 25 patients: In the first group (A) phaco prechop was performed; in the second group (B) divide and conquer was performed.

All surgeries were performed by the same surgeon in Kasr El Aini Hospital between January 2004 and August 2005 using the same phaco-machine (Geuder Megatron G 28101).

All patients chosen were above the age of 45 years. All eyes had no ocular pathology than the cataract without prior ocular surgery. Informed consent was obtained from every patient. Preoperative ocular assessment included: uncorrected visual acuity, best corrected visual acuity (BCVA), sit lamp biomicroscopy, applanation tonometer, fundus examination, B-scan ultrasound for dense cataract, ultrasonic biometry to determine the power of the posterior chamber intraocular lens (IOL), non-contact specular microscopy and pachymetry using non-contact Robo-7 Konan Incorporation specular microscope.

### Techniques

Surgeries were performed under general or local peribulbar anesthesia. A wire speculum was applied, a clear corneal incision was made, and one side port stab was made 90° from the main incision. A 5.5 mm continuous curvilinear capsulorrhexis (CCC) was made, followed by hydrodissection. The anterior chamber was then refilled with helon.

Group A cases were operated upon with the prechopping technique as follows:

*Karate Prechopping technique was used for nuclear cataract grade I and II as following:

- The sharp angled edge of the combo prechopper blade was inserted closed at the center of the nucleus by pushing downward. The blades are then opened slowly while pushing the nucleus downward.
- If unable to achieve bisection, the closed blades are inserted at the deepest portion of the nuclear crack, and reopened.
- In order to confirm complete nuclear division, the rounded side of the prechopper blade was used.
- Rotating the nucleus 90°, by pushing the distal end of the nucleus with the angled blade.
- Using the sharp and blunt blades of the combo prechopper alternately, each bisected nuclear fragment was divided in the same way.

*Counter prechopping technique was used for grade III nuclei as following:

- A Sinskey hook was inserted from the side port, passed just beneath the anterior capsulorrhexis edge to the central portion of the nucleus.
- While supporting the nucleus with the sustainer, the prechopper was inserted directly into the core of the nucleus.
- The prechopper was opened at the deepest portion of the nuclear body repeatedly until complete nuclear division is obtained.

Group B cases were operated upon using classic divide and conquer technique.

In both groups:

- The parameters of the machine were as follows: Vacuum: 200 mmHg, Ultrasound power: 60%, Ultrasound mode: linear, Aspiration: 22ml/minute, Infusion bottle height: 75 cm above the patient's head.
- The remaining cortical material was aspirated using a double way cannula.
- Refilling the anterior chamber and the capsular bag with helon, the incision was then enlarged to accommodate to a 5.5 mm polymethylmethacrylate intraocular lens. The helon was then aspirated and the incision was closed with 2 interrupted 2/10 nylon sutures, gentamycin and fortecorten were injected subconjunctivally at the conclusion of the surgery.

## Results

This study included 50 eyes of 50 patients divided randomly into 2 groups. Each group included 25 eyes: in the first group (A) phaco prechopping technique was performed; in the second group (B) divide and conquer technique was performed (Table 1).

**Table 1:** Grade of Nuclear Hardness in Groups A and B

	Group A	Group B
NI	7 (28%)	8 (32%)

NII	14 (56%)	13 (52%)

NIII	4 (16%)	4 (16%)

Group A included 11 males (44%) and 14 females (56%), the mean age of which was 56.76 years. Group B included 10 males (40%) and 15 females (60%), the mean age of which was 57.05 years.

The mean effective ultrasound time in group A was 19.36 seconds ± 8.51. In group B, the mean effective ultrasound time was 24.44 seconds ± 7.86 and the difference between the two groups was statistically significant (P = 0.033).

The mean preoperative endothelial cell count in group A was 2484.44 cells/mm^2^. In group B, the mean preoperative endothelial cell count was 2536.56 cells/mm^2^.

The mean endothelial cell count during the first postoperative week in group A was 2137.28 cells/mm^2^. In group B, the mean endothelial cell count during the first week postoperative was 2089.52 cells / mm^2^.

The mean endothelial cell count 3 months postoperative in group A was 2139.88 cells/mm^2^. In group B, the mean endothelial cell count three months postoperative was 2087.08 cells/mm^2^. The difference between the two groups was statistically insignificant (P = 0.558).

The mean endothelial cell loss three months postoperatively in group A was 344.56 (13.86%) which is statistically significant (P < 0. 05). In group B, the mean endothelial cell loss three months postoperatively was 449.48 (17.72%) that was statistically significant (P < 0.05). The difference in endothelial cell loss three months postoperatively between the two groups was statistically significant. (P = 0.001) (Table 2 and 3).

**Table 2:** Relation between Grade of Nuclear Hardness, Phaco Time, Total Energy, Endothelial Cell Count and Postoperative Endothelial Cell Loss in Both Groups

	Group	No of Eyes	Mean Effective Ultrasound Time (seconds)	Mean Total Energy (Joules)	Mean Preoperative Endothelial Cell Count cell/mm)	Mean Endothelial Cell count 12 Weeks Postoperative (cell/mm)	Endothelial Cell Loss 12 weeks Postoperative
NI	A	7	14.57	1559.14	2747.85	2426.14	321.71 (11.7%)
	B	8	21.62	2313.87	2650.12	2203.75	446.37 (16.84%)

NII	A	14	17.5	1872.5	2364.35	2039.42	324.92 (13.74%)
	B	13	22.38	2395.15	2491.46	2052.15	439.30 (17.63%)

NIII	A	4	34.25	3664.15	2443.75	1990.5	453.25 (18.54%)
	B	4	36.75	3932.25	2456	1967.25	488.75 (19.9%)

**Table 3:** Corneal Thickness in Both Groups Preoperative and Postoperative

	Preoperative	1st Week Postoperative	12 Weeks Postoperative
Group A	545.60 μ ± 38.60 μ	561.72 μ ± 38.55 μ	550.36 μ ± 39.20 μ

Group B	543.0 μ ± 25.47 μ	562.72 μ ± 34.93 μ	548.20 μ ± 26.13 μ

Four cases in group A (16%) and three cases in group B (12%) had localized posterior capsular rents that were discovered after completion of phacoemulsification; managed with anterior vitrectomy and in the sulcus posterior chamber intraocular lenses (PCIOL) implantation.

**Figure 1:**
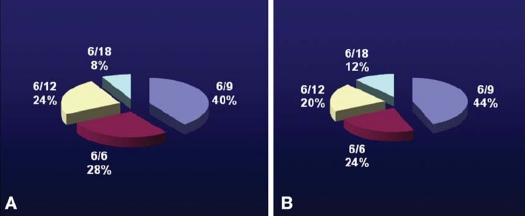
**(A)** Best corrected visual acuity 12 weeks postoperative in Group A; and **(B)** Group B.

Preoperative best corrected visual acuity in both groups were less than or equal to 6/36. Postoperative best corrected visual acuity in group B was 6/12 or better in 88% of cases as compared to 92% in group A with no statistical difference (Figure 1).

## Discussion

Phacoemulsification is associated with endothelial cell loss, but the percentage of loss may vary considerably. Endothelial cell loss during uneventful operations with preservation of the posterior capsule is due to intra-operative mechanical damage to the endothelium by heat production at the tip of the ultrasonic probe, ultrasound vibration, whirling lens cortical and nuclear fragments, irrigation solutions, air bubbles and surgical instruments.^4^

Phaco prechopping was introduced by Akahoshi in 1993 for dividing the nucleus without prior grooving, so that the total amount of ultrasound energy has been reduced with more protection of corneal endothelial cells.

The present study compared prechopping technique to the divide and conquer technique during a certain period of the learning curve of both techniques; that is first 25 cases in both groups performed by the same surgeon.

The mean effective ultrasound time in prechopping group was 19.36 seconds while in divide and conquer group it was 24.44 seconds. The ultrasound power in both groups was 60% in all cases. The mean total energy in prechopping group was 2071.52 Joules while in divide and conquer group it was 2615.08 Joules.

Therefore the ultrasound time was reduced in the prechopping group than the divide and conquer group by 20.78% with similar reduction in the total amount of ultrasound energy.

Our results are comparable to those reported by Auffarth and associates^5^ which was a standardized laboratory set up in 10 pairs of human autopsy eyes randomized for either the pre-chopper or the divide and conquer technique. They found that the average phaco-time with the Prechopper technique was 1.15±0.26min versus 1.48±0.28 min with the divide and conquer technique. The prechopping group had a 22.29% reduction in ultrasound time than the divide and conquer group.

Akahoshi^6^ reported that ultrasound time was reduced to less than 10% when compared with a standard divide-and-conquer technique, regardless of the grade of cataract. The lower ultrasound time reported by Akahoshi can be attributed partially to the more experience and the use of high vacuum techniques.

The results of this study were also comparable with the results of other techniques intended to reduce phaco time and energy utilized inside the eye.

Wong et al^7^ reported an effective phaco time of 50.4 seconds with the divide and conquer technique compared to 17.4 seconds with phaco chop. Pierazzoli et al^8^ reported an effective phaco time of 25.53 seconds with phaco chop as compared to 87.26 seconds with divide and conquer.

Other techniques were developed to reduce phaco time and total energy. In short groove nucloefractis by Abdelrahman,^9^ the effective phaco time was 9.1 seconds in moderately hard nuclei and 15.8 seconds in harder nuclei. In phaco one chop nucleotomy by Sebban,^10^ the mean effective phaco time was found to be 8.3, 2.0, and 1.0 for high, moderate and low nuclear hardness respectively. These low results as compared to the present study may be attributed to the more experience of the surgeons and the high vacuum settings in one chop nucleotomy technique.

In Our study, the mean endothelial cell loss three months postoperatively in prechopping group was 344.56 (13.86%) cells/mm^2^, and in divide and conquer group it was 449.48 (17.72%) cells/mm^2^.

Comparative studies evaluating the percentage of cell loss after phacoemulsification have been reported. Kosrirukvongs and associates^11^ reported a mean endothelial cell loss of 10.7% three months following four quadrants divide and conquer phaco fracture. It was also noted that in both groups, the higher the grade of nuclear hardness, the longer the ultrasound time, the more the energy utilized and the higher the endothelial cell loss.

In grade I nuclear cataract, the phaco time in group A was less than that of group B, however it was statistically insignificant. In grade II nuclear cataract, the phaco time in group A was both clinically and statistically significant.

The relatively high rate of posterior capsular tears in both groups was related to the learning curve. Group A cases had a higher rate of posterior capsular tears compared to divide and conquer technique with no statistical difference; this could be attributed to the further unfamiliarity with the technique and instruments.

In conclusion, both prechopping and the divide and conquer techniques were suitable for phaco beginners with comparable results and complications. However the former technique utilized less phaco time and energy without significant effect on the final surgical outcome.

## References

[1] Gimbel HV (1991). Divide and conquer nucleofractis phacoemulsification—development and variations. J Cataract Refract Surg.

[2] Akahoshi T (2001). Phaco Prechop: Mechanical Nucleofracture Prior to Phacoemulsification. The Frontier of Ophthalmology in the 21st Century.

[3] Akahoshi T (1998). Phaco prechop: Manual nucleofracture prior tophacoemulsification. Op Tech Cataract Ref Surg.

[4] Kohlhaas M (1997). Klemm M, Kammann J and Dichard G: Endothelial cell loss secondary to two different phacoemulsification techniques. Ophthalmic Surg Lasers.

[5] Auffarth G, Peng Q, Daines B (2000). Comparison of pre-chopper vs. divide & conquer phacoemulsification techniques: A standardized laboratory study, 98^th^ Annual Meeting DOG.

[6] Akahoshi T (2002). The karate prechop technique, cataract and re-fractive surgery today.

[7] Wong T, Hingorani M, Lee V (2000). Phacoemulsification time and power requirements in phaco chop and divide and conquer nucleofractis techniques. J Cataract Refract Surg.

[8] PierazzoIi G, D'Eliseo D, Ziosi M, Acciarri R (1996). Effects of phacoemulsification time on the corneal endothelium using phaco fracture and phaco chop techniques. Journal of Cataract and Refractive Surgery.

[9] Abdelrahman AM (2003). Short Groove Nucleofractis: A safe Method of Nucleus Disassembly, Bull. Ophthalmol. Soc. Egypt.

[10] Sebban I (2002). Phaco one-chop nucleotomy. J Cataract Refract Surg.

[11] Kosrirukvonss P, Slade SG, Berkeley RG (1997). Corneal endothelial changes after divide and conquer versus chip and flip phacoemulsification. J Cataract Refract Surg.

